# Comparative Efficacy of Neuromodulation and Structured Exercise Program on Autonomic Modulation in Fibromyalgia Patients: Pilot Study

**DOI:** 10.3390/jcm13154288

**Published:** 2024-07-23

**Authors:** Alejandro Rubio-Zarapuz, María Dolores Apolo-Arenas, Orlando Fernandes, José Francisco Tornero-Aguilera, Vicente J. Clemente-Suárez, Jose A. Parraca

**Affiliations:** 1 Faculty of Sports Sciences, Universidad Europea de Madrid, Tajo Street, s/n, 28670 Madrid, Spain; alejandro.rubio@universidadeuropea.es (A.R.-Z.); josefrancisco.tornero@universidadeuropea.es (J.F.T.-A.); vicentejavier.clemente@universidadeuropea.es (V.J.C.-S.); 2 Department of Medical Surgical-Therapy, Faculty of Medicine and Health Sciences, Universidad de Extremadura, 06006 Badajoz, Spain; mdapolo@unex.es; 3 Research Group PhysioH, University of Extremadura, 06006 Badajoz, Spain; 4 Departamento de Desporto e Saúde, Escola de Saúde e Desenvolvimento Humano, Universidade de Évora, 7004-516 Évora, Portugal; orlandoj@uevora.pt; 5 Comprehensive Health Research Centre (CHRC), University of Évora, 7004-516 Évora, Portugal; 6 Grupo de Investigación en Cultura, Educación y Sociedad, Universidad de la Costa, Barranquilla 080002, Colombia

**Keywords:** fibromyalgia, neuromodulation therapy, exercise, autonomic modulation, heart rate variability, cortical arousal

## Abstract

**Background:** Fibromyalgia is a chronic disorder marked by widespread muscle and joint pain, persistent fatigue, sleep disturbances, and irregularities in the autonomic nervous system (ANS). **Methods:** This study compared the effectiveness of neuromodulation using the EXOPULSE Mollii suit with a structured exercise program in regulating ANS function in fibromyalgia patients. In this randomized, longitudinal crossover study, 10 female patients were randomly assigned to either the Suit + Exercise group or the Exercise + Suit group. Each group participated in two sessions per week for eight weeks, followed by a two-week washout period before switching to the other intervention. We measured cortical arousal, microcirculation, and heart rate variability (HRV) before and after the 1st, 8th, and 16th sessions. **Results:** The results showed significant improvements in cortical arousal, HRV, and microcirculation with the neuromodulation treatment whereas the exercise program only produced short-term improvements in cortical arousal. **Conclusion:** The EXOPULSE Mollii suit exhibited cumulative benefits on ANS modulation over time, suggesting potential long-term advantages for managing fibromyalgia. However, further research is needed to explore the delayed effects of both treatments on ANS modulation.

## 1. Introduction

Fibromyalgia is a long-term disorder characterized by widespread muscle and joint pain, persistent fatigue, sleep issues, irregularities in the autonomic nervous system (ANS), cognitive issues, heightened sensitivity, and various physical and mental health comorbidities [1,2]. It ranks among the top three most prevalent musculoskeletal disorders worldwide, following lower back pain and osteoarthritis, affecting approximately 2–3% of the global population [2]. This prevalence increases to roughly 4.7% in Western Europe [3]. Fibromyalgia primarily affects women, with a ratio of three females to every one male [4], and its prevalence increases with age, reaching its highest incidence between 50 and 60 years [5].

Fibromyalgia-related pain varies widely, influenced by factors such as occupational demands, comorbid health conditions, and environmental conditions like weather changes or stress [6]. Patients often endure both physical and mental fatigue, ranging from mild tiredness to severe fever-like exhaustion [7]. Additional symptoms include insomnia [7,8], cognitive impairments such as memory loss [9], depression and anxiety [10], headaches [11], digestive disturbances [12], urinary issues [13], morning stiffness [14], and autonomic imbalances like dry eyes, dry mouth, blurred vision, and light sensitivity [2]. Emotional distress and negative feelings are also prevalent, contributing to an increased incidence of mental disorders; for instance, up to 60% of patients may experience anxiety while depression rates range from 14% to 36%, compared to 6.6% in the general population [15]. An imbalance in the ANS is a hallmark feature of fibromyalgia, leading to irregular heart rhythms, gastrointestinal disturbances, and abnormal blood pressure fluctuations [16]. This dysregulation significantly contributes to the pervasive chronic pain and fatigue characteristic of fibromyalgia, exacerbating other symptoms like sleep disruptions and temperature sensitivity [17].

Diagnosing fibromyalgia is challenging due to the lack of unique biomarkers and its non-specific symptoms, which differentiate it from other rheumatic conditions [18]. Over the past three decades, five diagnostic criteria have been established [19]. Although the precise etiology of fibromyalgia remains unknown, it is believed to involve genetic factors, significant psychological stress, peripheral inflammation, and disruptions in central pain processing leading to nociplastic pain [20]. Advances in microRNA, proteomic, and metabolomic studies have shown promise in improving disease identification [21].

Managing fibromyalgia necessitates a multidisciplinary approach encompassing medication, psychological support, patient education, physical activity, and dietary modifications [22]. Pharmacological treatment primarily focuses on pain relief, with central-nervous-system-active medications like antidepressants and anticonvulsants proving effective in altering pain pathways and reducing nervous system excitability [23]. However, only about 25% of patients achieve a 30% reduction in symptoms with antidepressant therapy [24]. Treatment strategies vary widely, ranging from muscle relaxants [25,26,27], pain relievers [28,29], sleep aids, psychiatric medications [29,30], and cannabinoids [31]. Nevertheless, no single drug consistently benefits more than half of the treated patients [32]. Cognitive–behavioral therapy offers promise in managing pain and improving mood and physical functioning more effectively than other methods [33]. Patient education plays a crucial role in fostering an understanding of the chronic condition and promoting proactive management [34,35,36].

Non-pharmacological therapies provide broader comprehensive benefits that are challenging to achieve with medication alone [37]. Treatments such as spa therapy [38,39], Tai Chi, Qigong, and yoga [40,41,42], alongside mindfulness [43,44], hypnosis [45], acupuncture [46], thermal or cryotherapy [39], hyperbaric oxygen therapy [47], and various brain stimulation forms [48,49], have been explored. Given the pervasive impact of ANS dysregulation on daily functioning, it remains a key target for intervention. Therapeutic approaches like biofeedback, yoga, and structured exercise programs not only alleviate symptoms but also aim to restore autonomic balance [50,51]. Neuromodulation techniques, including localized transcutaneous electrical nerve stimulation (TENS) [52,53,54] and the EXOPULSE Mollii^®^ suit [55,56,57], have demonstrated positive effects on pain perception, muscle oxygen levels, parasympathetic activity, and overall functionality. Exercise and dietary interventions are often recommended before pharmacological treatments, emphasizing aerobic and strength training, weight management, and dietary modifications [2,29]. These practices enhance posture, reduce inflammation related to obesity, and improve pain management and functionality [58]. A single strength training session has demonstrated to markedly enhance breathing, pain perception, brain activity, muscle oxygenation, autonomic regulation, and physical performance.

This study aimed to investigate the impact of 16 treatment sessions with the EXOPULSE Mollii suit compared to a conventional 16-session exercise training regimen on autonomic modulation in fibromyalgia patients. The central hypothesis posited significant differences in autonomic modulation between pre- and post-treatment evaluations across the treatment modalities.

## 2. Materials and Methods

### 2.1. Ethical Aspects

This research followed the ethical guidelines outlined in the Declaration of Helsinki. The intervention received formal approval from the University of Évora’s Research Ethics Committee (approval number 22033, dated 31 January 2022). Before the study began, the objectives and procedures were clearly explained to all participants, who then voluntarily signed an informed consent form, indicating their understanding and willingness to participate.

### 2.2. Study Design

This research was conducted at the Faculty of Medicine, University of Badajoz, Spain. It employed a randomized, crossover, longitudinal, and experimental design to assess and contrast the long-term effects of two different therapeutic treatments on patients diagnosed with fibromyalgia, strictly adhering to the 2016 American College of Rheumatology (ACR) criteria [15].

The therapeutic approaches evaluated included a regimen involving the use of the EXOPULSE Mollii suit (Exoneural Network AB, Solna, Sweden) and a structured physical exercise protocol. Participant recruitment occurred between September 2022 and December 2022, providing a comprehensive and thorough inclusion phase. The intervention phase commenced in January 2023 following a meticulous screening and selection process to ensure that every patient met the stringent diagnostic criteria for fibromyalgia as specified by the ACR. The study’s methodological framework was deliberately designed to meticulously evaluate and document the immediate, short-term, and long-term outcomes of these therapeutic interventions.

### 2.3. Participants

Following an exhaustive recruitment and evaluation phase, the study successfully enrolled a total of 10 female patients diagnosed with fibromyalgia (mean age: 51.6 ± 7.18 years; mean weight: 68.5 ± 8.26 kg; mean height: 160 ± 3.80 cm; Body Mass Index (BMI): 26.7 ± 2.79 kg/m^2^). Eligibility criteria required participants to meet several stringent conditions: participants had to be formally diagnosed with fibromyalgia by a rheumatologist based on the ACR criteria, with the diagnosis established at least three months prior to the study. Additionally, the study was restricted to females aged 18–67 who could walk independently without assistive devices. Exclusion criteria were meticulously defined. Candidates were excluded if they were concurrently involved in other clinical investigations, had previously engaged in neuromodulation therapy or taken part in organized exercise regimens within the six months preceding the study, did not provide written consent, had concurrent neurological disorders or conditions significantly affecting pain perception, had undergone recent surgical procedures or sustained musculoskeletal injuries within the past six months, had severe cardiovascular or respiratory conditions contraindicating exercise, or were undergoing opioid therapy or had had changes in their pain management medication in the past month. The final participant pool represented the maximum feasible cohort size given the scheduling opportunities, facility capacities, and available personnel resources. This meticulous selection process ensured that the participant group adhered strictly to both operational and clinical criteria, enabling a controlled and methodologically sound evaluation of the intervention’s impact on fibromyalgia symptoms.

### 2.4. Intervention

To achieve the aims of this study, participants were randomly assigned to one of two groups using a computer-generated randomization sequence to ensure unbiased allocation: the Suit + Exercise group or the Exercise + Suit group. The experimental design ensured procedural consistency across both groups. Participants underwent two sessions of their assigned intervention per week for a duration of 8 weeks, resulting in a total of 16 sessions per intervention. Following these sessions, a 2-week washout period was implemented to eliminate any residual effects of the treatments, helping restore participants’ physiological baselines and ensure the integrity of subsequent results. After this interval, a second sequence of 16 sessions was conducted, with participants receiving the alternative intervention to complete the crossover design. In the initial phase, the Suit + Exercise group underwent neuromodulation treatment before proceeding to the exercise protocol. In contrast, the Exercise + Suit group began with the Exercise intervention and then received neuromodulation therapy. The treatments administered are described as follows:

- Suit: Participants underwent a 60 min session with the EXOPULSE Mollii suit, an advanced medical device designed to alleviate spasticity and improve motor function in individuals with neuromuscular disorders equipped with 58 embedded electrodes that deliver low-frequency electrical stimulation to specific muscle groups at an intensity of 2 milliamperes (mA) and a pulse width of 30 milliseconds (ms). This targeted both agonist and antagonist muscles to promote muscle relaxation and enhanced movement control with the capacity of engaging specific muscle groups. In this treatment, all 58 electrodes were active during treatment. This protocol followed the treatment procedures outlined in prior studies [55,56,59,60]. Each participant was assisted by a certified professional to ensure accurate electrode placement. Once the suit was correctly applied and the control unit attached, the participant was positioned supine on a massage table, at which point the suit was activated to commence the session.

**Figure 1:**
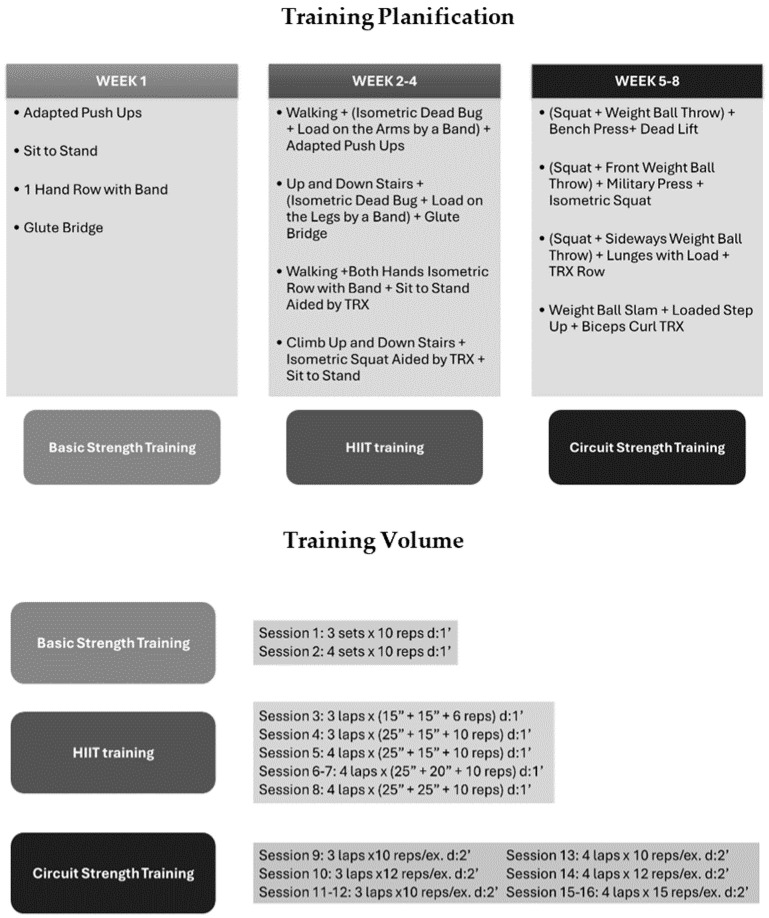
Exercise Intervention. ′: minutes; ″: seconds; reps/ex.: repetitions per exercise.

Exercise: Participants engaged in a 1 h training session per meeting, starting with a mobility warm-up, followed by a main training session involving strength exercises and High-Intensity Interval Training (HIIT). This regimen was systematically progressed throughout the intervention period, as detailed in Figure 1. The intervention was divided into three blocks: 1st block comprised sessions 1 and 2, characterized by strength training, which was integrated as a familiarization phase, which was crucial for establishing a foundation for subsequent intensive training. This phase aimed to acclimate participants to the exercise regimen, ensuring their comfort with the routines and minimizing the risk of injury. Then, the 2nd block formed by sessions from 3rd to 8th was focused in HIIT training; after warming up, all sessions were composed of 4 circuits each participant had to complete in the same session with an incremental training volume throughout the block. Finally, the 3rd block was composed of strength training with a circuit format; participants performed all exercise in the same circuit before resting to complete a lap after completing all laps in the circuit, then passed to the successive circuit until completion of the session.

### 2.5. Measurements

Outcome measures were meticulously evaluated both prior to and following the interventions at three critical junctures: the 1st, 8th, and 16th treatment sessions for each treatment modality. This multistage evaluation strategy aligns with methodologies previously validated in longitudinal research, providing a robust framework for quantifying the intervention effects [55,56].

#### 2.5.1. Thermography

**Figure 2:**
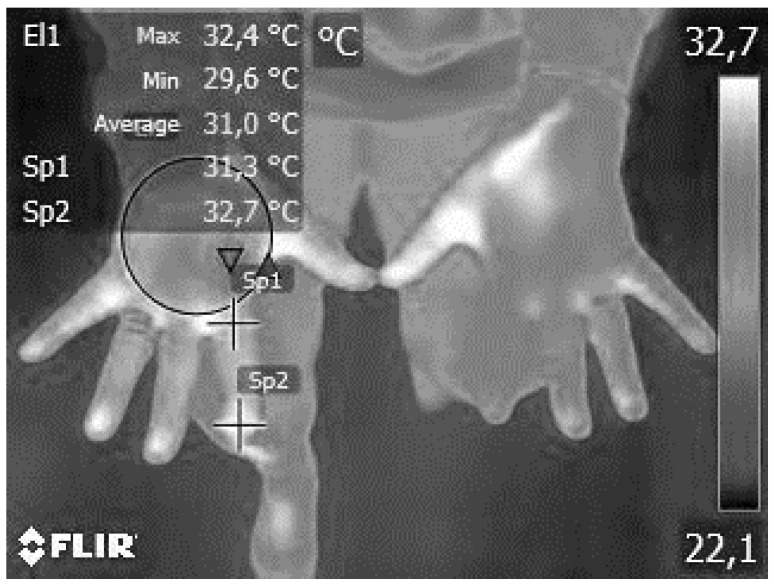
Thermal imaging assessment using the FLIR E8-XT system.

Thermal imaging of the specified hot points was performed using the advanced FLIR E8-XT thermography system (FLIR Systems Inc., Wilsonville, OR, USA) (Figure 2) [61]. This procedure entailed obtaining two accurate measures of the dorsal (back) and palmar (front) aspects of the hand, following methodologies from previous research [61]. The FLIR E8-XT system offered an automated and precise evaluation of thermal anomalies, facilitating the detection of subtle variations in skin temperature that were indicative of underlying physiological processes.

#### 2.5.2. Cortical Arousal

**Figure 3:**
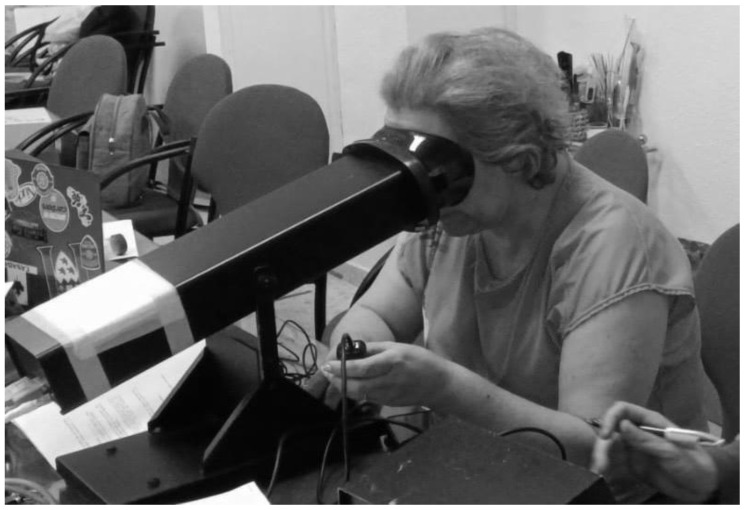
Cortical arousal assessment using the Lafayette Instrument Flicker Fusion Control Unit.

Cortical arousal was measured using the Critical Flicker Fusion Threshold (CFFT) technique, performed in a controlled viewing chamber using the Lafayette Instrument Flicker Fusion Control Unit Model 12,021 (Lafayette Instrument Company, Lafayette, IN, USA) (Figure 3). This method complied with previously established guidelines [62,63], guaranteeing methodological uniformity and accuracy. The CFFT technique entails identifying the highest frequency at which a person perceives a flickering light as continuous, reflecting the brain’s processing speed and overall cortical arousal.

#### 2.5.3. HRV Variables

Heart Rate (HR) and Heart Rate Variability (HRV) were measured using a Polar H10 chest band (Polar Electro, Kempele, Finland), which operates with a sampling frequency of 1000 Hz, capturing both the number of beats per minute for HR analysis as well as RR intervals (time interval between R waves on the electrocardiogram) for HRV analysis. HRV data were examined using Kubios HRV v2.2 software (Kubios Oy, Kempele, Finland), with no correction factor applied as the obtained measurements were clear and free of noise. The subsequent HRV variables were examined [64]:

- Time-Domain Analysis: RMSSD (Root Mean Square of Successive Differences) reflects beat-to-beat variance in HR, primarily estimating vagally influenced variations in HRV and PNN50%, the percentage of adjacent NN intervals differing by more than 50 ms, closely correlating with parasympathetic nervous system (PNS) activity.
- Frequency-Domain (Spectral) Measures: Low-Frequency (LF) Power Component (0.04–0.15 Hz) and High-Frequency (HF) Power Component (0.15–0.40 Hz) in normalized units (n.u.).
- Nonlinear Domain Analysis: SD1 reflects parasympathetic activity via a Poincaré plot on the transverse axis and SD2 indicates long-term variations in RR intervals and serves as an inverse indicator of sympathetic activity.

These variables provide a comprehensive assessment of autonomic modulation by evaluating both time and frequency domains as well as nonlinear dynamics of HRV.

### 2.6. Statistical Analysis

SPSS (Statistical Package for the Social Sciences, version 25, IBM, Armonk, NY, USA) was used for statistical analysis. Descriptive statistics are presented as means ± SDs. The normality distribution of the data was evaluated with the Kolmogorov–Smirnov test. *T*-tests were applied to evaluate the intervention effects on parametric variables while the Wilcoxon test was used for non-parametric variables to evaluate differences between pre- and post-session measurements in measured sessions (1st, 8th, and 16th) as well as between different session measurements. For in-between-group comparisons, a one-way ANOVA was conducted. The significance level was set at *p* ≤ 0.05 for all tests. Effect sizes were calculated using epsilon squared (ε^2^) for ANOVA, rank of biserial correlation for the Wilcoxon test, and Cohen’s d for *t*-tests to offer a comprehensive understanding of the intervention effects. This metric measures the difference between two means in terms of standard deviation, aiding in the interpretation of practical significance. According to Cohen’s standards, effect sizes are classified as small (d = 0.2), medium (d = 0.5), and large (d = 0.8). These benchmarks were used to assess the size of treatment effects observed in this study, ensuring comprehension of their clinical relevance.

## 3. Results

Table 1 and Table 2 display descriptive statistics for all variables measured before and after the 1st, 8th, and 16th sessions in both treatment groups. Cortical arousal is a critical indicator of the central nervous system’s response to interventions. Our results demonstrate that both the EXOPULSE Mollii suit and structured exercise significantly improved cortical arousal over the course of the study. In the Suit intervention group, cortical arousal increased incrementally by 0.5 after the 1st session, 1.1 after the 8th session, and 1.4 after the 16th session. These findings suggest a steady cumulative effect of the neuromodulation treatment, indicating its potential to enhance central nervous system activity over time. Conversely, the Exercise intervention group showed a more immediate but less consistent increase in cortical arousal, with values rising by 1.5 after the 1st session, 1.2 after the 8th session, and 3.8 after the 16th session. Although the exercise program produced a higher initial increase in cortical arousal, the fluctuations observed suggest that the benefits might not be as sustainable as those observed with the Suit intervention. In terms of thermography variables, a decrease was observed in all measures after each session in the Suit group: palm (0.6, 0.4, 1.5), backhand (0.1, 0.2, 1), distal index (0.1, 1, 4.2), and proximal index (0.6, 1.2, 2.2). Conversely, the Exercise group exhibited an increase in thermography measures after each session: palm (2.2, 1.4, 1.3), backhand (1.9, 1.3, 0.8), distal index (2.7, 1.6, 2), and proximal index (3, 1.3, 1.5). Heart rate (HR) values showed varying trends across sessions. In the Suit group, there was an increase in mean HR values in the 1st (5.63) and 8th (2.8) sessions, followed by a decrease in the 16th session (−2.9). In the Exercise group, there was a consistent increase across all three sessions: 2.08, 7.88, and 12.28. Maximum and minimum HR values also differed between the interventions. In the Suit group, maximum HR decreased (7.06, 1.56, 3.7), as did minimum HR (4.02, 3.29, 1.25). In contrast, the Exercise group saw increases in maximum HR (4.19, 12.74, 13.27) and minimum HR (0.47, 5.44, 10.25). HRV is a measure of autonomic nervous system function and balance between sympathetic and parasympathetic activity. Our study found that the EXOPULSE Mollii suit significantly improved HRV, indicating enhanced parasympathetic activity and better autonomic balance. Before the intervention, the baseline HRV was relatively low, reflecting the typical autonomic dysregulation in fibromyalgia patients. After the 16th session, the Suit intervention group showed marked improvements in HRV, suggesting that neuromodulation could effectively restore autonomic balance. In contrast, the Exercise intervention group did not exhibit significant changes in HRV, highlighting a potential limitation of exercise alone in addressing autonomic dysregulation in fibromyalgia patients. These findings underscore the importance of exploring complementary therapies to achieve optimal autonomic regulation. RMSSD increased in the Suit group (1.15, 1.32, 0.03) but decreased in the Exercise group (1.93, 3.79, 3.05). PNN50 showed a decrease in the 1st session (0.11) and increases in the 8th (2.39) and 16th (0.39) sessions in the Suit group while it consistently increased in all Exercise sessions (0.61, 0.69, 9.54). In terms of frequency-domain measures, the high-frequency (HF) component decreased in both interventions (Suit: 5.57, 3.11, 3; Exercise: 7.81, 7.06, 0.4) while the low-frequency (LF) component increased in both interventions (Suit: 5.57, 4.61, 3.03; Exercise: 7.83, 7.1, 0.43). The nonlinear domain analysis showed that SD1 increased in the Suit group (0.82, 0.94, 0.02) but decreased in the Exercise group (1.37, 2.68, 2.23). SD2 increased in all sessions in the Suit group (1.65, 5.76, 4.61) while, in the Exercise group, it increased in the 1st session (2.39) but decreased after the 8th (3.11) and 16th (4.21) sessions.

Table 3 present the comparative statistics of the study’s findings. Table 3 displays the results of the one-way ANOVA. Significant differences were observed in palm, backhand, and proximal index finger temperatures both before and after the intervention in the Suit treatment, and in the Exercise treatment, except for the proximal index finger post-intervention measurements. Additionally, significant differences in mean HR were found in the pre-intervention measurements for the Suit intervention and in the post-intervention measurements for the Exercise treatment. Maximum and minimum HR values showed significant differences in post-intervention measurements for the Exercise treatment. HRV measurements indicated significant differences in HF and LF values in the pre-intervention measurements for the Exercise treatment. Comparisons between treatments in Table 4 also highlights significant differences in palm, distal, and proximal index finger temperatures after the 1st session. After the 8th session, significant differences were found in palm, distal, and proximal index finger temperatures, as well as in the minimum HR, RMSSD, PNN50, and SD1. Following the 16th session, significant differences were identified in distal and proximal index finger temperatures and minimum HR.

Table 5 illustrates the *t*-test results comparing baseline and post-session measurements for each treatment. In the Suit treatment, significant differences were found in the mean HR in the 1st session, cortical arousal, the minimum HR, and SD2 in the 8th session and cortical arousal and distal index finger temperature in the 16th session. In the Exercise treatment, significant differences were identified in cortical arousal; palm, backhand, and proximal index finger temperatures; HF, and LF in the 1st session; cortical arousal, palm, and proximal index finger temperatures; mean, maximum, and minimum HR; and RMSSD, PNN50, and SD1 in the 8th session; and distal and proximal index finger temperatures; mean, maximum, and minimum HR; and RMSSD in the 16th session.

Table 6 presents *t*-test results between different session measurements in the Suit intervention. Significant differences were found in cortical arousal between the 8th pre-session and 16th post-session measurements; in palm temperature pre-session measurements between the 1st and 8th sessions, the 8th and 16th sessions, and the 1st and 16th measurements; and between the 1st and 16th post-session measurements. For backhand temperature, significant differences were observed between the 1st and 8th sessions, the 8th and 16th sessions, and the 1st and 16th pre-session measurements and between the 1st pre-session and 16th post-session measurements. For distal index finger temperature, significant differences were found between the 8th and 16th pre-session measurements and the 1st and 16th pre-session measurements. For proximal index finger temperature, significant differences were found between the 1st and 8th, 8th and 16th, and 1st and 16th pre-session measurements. The mean HR showed significant differences between the 8th and 16th pre-session measurements, and HF and LF showed significant differences between the 8th and 16th post-session measurements.

Table 7 shows the *t*-test results for different session measurements in the Exercise intervention. Meaningful variations were identified when comparing the 8th and 16th pre-session measurements in the mean and maximum HR, RMSSD, HF, LF, and SD1; between the 1st and 16th pre-session measurements in palm, backhand, and proximal index finger temperatures; between the 1st and 8th post-session measurements in palm temperature; and between the 8th and 16th post-session measurements in proximal index finger temperature; mean, maximum, and minimum HR; RMSSD; and SD1. Further differences were observed between the 1st and 16th post-session measurements in backhand and proximal index finger measurements; mean, maximum, and minimum HR; RMSSD; and SD1. Additionally, meaningful variations were identified between the 1st pre-session and 8th post-session measurements in palm, backhand, and proximal index finger temperatures; RMSSD; HF; LF; and SD1 and between the 1st pre-session and 16th post-session measurements in palm, backhand, and proximal index finger temperatures; mean, maximum, and minimum HR; RMSSD; HF; LF; and SD. Finally, there were meaningful variations between the 8th pre-session and 16th post-session measurements in palm, backhand, proximal, and distal index finger temperatures; the mean, maximum, and minimum HR; RMSSD; HF; LF; and SD1.

These comparative analyses provide critical insights into the differential impacts of neuromodulation and structured exercise programs on autonomic modulation in fibromyalgia patients.

## 4. Discussion

Fibromyalgia often involves the dysregulation of the ANS, with a greater inclination towards the sympathetic branch compared to the parasympathetic branch. This imbalance results in a constant “fight or flight” response, leading to general inflammation, exacerbating pain, and disturbing sleep among other effects. Therefore, finding a treatment that could help correct ANS modulation is crucial. This research aimed to explore the effects of two different treatments on ANS modulation. Our findings confirmed the initial hypothesis, showing significant differences in ANS modulation after treatment. However, these differences varied depending on the type and phase of the treatment.

Neuromodulation with the EXOPULSE Mollii suit primarily targets the central nervous system by delivering low-frequency electrical stimulation to specific muscle groups, which can enhance muscle relaxation and improve blood circulation. This electrical stimulation may also modulate pain pathways and reduce muscle stiffness, potentially leading to improved autonomic balance [59]. The suit’s stimulation of both agonist and antagonist muscles could promote a more balanced activation of the sympathetic and parasympathetic branches of the ANS. Over time, repeated sessions of neuromodulation could lead to cumulative benefits, as observed in the increased cortical arousal and improved HRV metrics. These effects suggest that neuromodulation may help reset the autonomic tone towards a more parasympathetic-dominant state, reducing the overall stress load on the body [55]. Cortical arousal allows for the measurement of a patient’s cortical processing abilities at a given moment. A lower value on this variable indicates greater critical processing capability, sympathetic activation, and a higher “fight or flight” response while a higher value indicates lower cortical processing capacity and greater parasympathetic activity. Previous studies did not find statistically significant differences in cortical arousal after a single 60 min neuromodulation session with the EXOPULSE Mollii suit [56], which aligned with our results for the 1st session of treatment. However, there were significant differences in the 8th and 16th sessions, with 1.1 and 1.4 increases in cortical arousal values, indicating greater parasympathetic activation after treatment for both sessions. These values show a cumulative increase in cortical arousal effects with successive neuromodulation sessions, corroborated by the effect sizes, which increased from 0.73 in the 8th session to 0.97 in the 16th session, both being large effect sizes. Furthermore, in the Exercise treatment, a statistically significant increase was observed after the 1st and 8th sessions, with respective effect sizes of 1 and 0.7. Similar findings have been reported in previous research, which found significant differences in cortical arousal after a single strength exercise session [56]. The behavior of this variable in the Exercise treatment contrasted with that in the Suit treatment as the effect sizes in the Exercise intervention showed a decreasing trend. This could be due to the increasing intensity and introduction of HIIT throughout the program, which produced greater sympathetic activation during the session. Thus, a strength-focused session could yield more benefits for fibromyalgia patients in terms of cortical arousal. However, these findings are based on measurements taken immediately after an exercise session. A second measurement after several hours had passed might have shown a compensation of the ANS with a greater parasympathetic tone, which should be considered in future research. Therefore, these findings suggest that exercise treatment can benefit cortical arousal in the short term although the effects diminish as sessions proceed. In contrast, the Suit intervention showed no significant effects in the short term but demonstrated cumulative effects on cortical arousal as treatment progressed.

Exercise, particularly when involving strength and HIIT, has been shown to have significant effects on autonomic modulation. Exercise induces a temporary increase in sympathetic activity to meet the physical demands placed on the body, followed by a post-exercise recovery period characterized by increased parasympathetic activity. This pattern helps enhance overall autonomic flexibility [65]. Regular physical activity improves cardiovascular fitness, enhances vagal tone, and reduces the resting heart rate, contributing to better HRV. Additionally, exercise can reduce systemic inflammation and improve endothelial function, which may further support autonomic balance. The immediate sympathetic activation observed during exercise sessions may be beneficial in ‘resetting’ autonomic control mechanisms while the recovery phase promotes parasympathetic dominance [66]. Microcirculation can also be used to measure ANS modulation. Higher microcirculation in the periphery, indicated by higher temperature in the extremities, can be translated to greater sympathetic modulation while lower temperature indicates greater parasympathetic activation. The results show no significant differences among any thermography measures in the Suit intervention, except for the distal index finger temperature in the 16th session, which suggests greater parasympathetic modulation after this session, with a large effect size of 1.4. These findings contrast with previous research that found significant differences after a single neuromodulation session in palm temperature but no significant differences in distal index finger temperature [56]. Additionally, significant differences were found between baseline measurements taken in each session, showing that these variables can be affected by the environment. This underscores the need to combine thermography with other spectrum variables to accurately measure ANS modulation. In addition, significant differences were also found between the 1st baseline and 16th post-session measurement, showing that neuromodulation effects on microcirculation progressively increase as neuromodulation treatment progresses. In contrast to previous findings [56], there were significant differences for the Exercise intervention in palm, backhand, and proximal index finger temperatures in the 1st session, with corresponding effect sizes of 0.82, 0.77, and 0.95; in the 8th session, significant differences were found in palm and proximal index finger temperatures, with effect sizes of 0.86 and 0.88, respectively; and in the 16th session, significant differences were found in distal and proximal index finger temperatures, with effect sizes of 0.92 and 0.5, respectively. These results show that the effects on microcirculation induced by exercise are greater than those produced by the suit intervention but are also more variable. Significant differences between different session measurements further highlight the variability of microcirculation depending on the environment, exercise type, and modality. Comparing the effects of the Suit and Exercise interventions on microcirculation reveals a clear difference: temperature decreased from baseline to post-intervention measurements in the Suit group while it increased in the Exercise group. This indicates a potential parasympathetic modulation effect in the neuromodulation treatment while suggesting an immediate sympathetic modulation effect in the Exercise group, induced by the need for a greater sympathetic tone to cope with the physical effort of the exercise session. This necessitates greater blood flow to the muscles and a subsequent increase in body temperature, explaining the greater microcirculation measured. As with cortical arousal, it would be interesting to measure this variable several hours after an intervention to see whether there is a compensation in microcirculation.

HRV is the variable most commonly used to measure ANS modulation. In this study, significant differences were found only in the mean HR after the 1st session, with a lower value and a corresponding large effect size of 0.84, which can be translated to greater parasympathetic activation. Moreover, the minimum HR and SD2 after the 8th session were also significantly different, with the HR having a lower value (effect size of 0.92) and SD2 having a greater value (effect size of 0.96), both indicating greater parasympathetic branch activation. However, as no previous studies using the EXOPULSE Mollii suit have measured these variables, further research on the effects of this tool on HRV is necessary. Further on, the Exercise intervention produced significant differences in HF and LF values in the 1st session; mean, maximum, and minimum HR; RMSSD; PNN50; and SD1 in the 8th session; and mean, maximum, and minimum HR and RMSSD in the 16th session, with very large effect sizes. These variations suggest an immediate shift towards a sympathetic tone in ANS modulation. The effects of exercise therapy on HRV in fibromyalgia remain unclear as some evidence suggests no beneficial effects on HRV values [67] while other evidence indicates beneficial effects on these variables [68,69]. Our findings suggest an immediate increase in sympathetic tone after the exercise session, which could later be compensated by an enhanced parasympathetic-dominant ANS modulation, leading to beneficial effects from this treatment. Therefore, future research should aim to measure HRV several hours after an exercise session to determine whether delayed HRV effects occur.

In conclusion, neuromodulation treatment with the EXOPULSE Mollii suit holds promise for fibromyalgia patients’ ANS modulation, enhancing the parasympathetic branch with cumulative effects that grow as treatment progresses. However, further research is needed as this is a novel treatment that requires more exploration. The Exercise intervention focused on strength and HIIT training showed beneficial immediate and short-term effects on cortical arousal when measured right after the intervention. Future research should investigate the neuromodulation effects of exercise on fibromyalgia several hours after treatment as they may show a delayed and significant effect in this patient population.

### 4.1. Practical Applications

This research demonstrates that neuromodulation with the EXOPULSE Mollii suit benefits neuromodulation in fibromyalgia patients, leading to incremental improvements in cortical arousal over time, as well as to positive effects on HRV and microcirculation. Additionally, exercise treatment has shown beneficial effects on cortical arousal although these effects are limited to the immediate and short-term periods. However, no significant beneficial effects were observed in HRV and microcirculation immediately after the Exercise intervention.

### 4.2. Limitations of the Study

The primary limitation of this research study was the small sample size, stemming from the characteristics of this patient population, which made recruitment challenging due to the extended commitment required from participants. Additionally, the absence of in-treatment HRV measurements limited our understanding of the ANS modulation response to these treatments. HRV bands were not used during neuromodulation sessions with the EXOPULSE Mollii suit as they could interfere with treatment according to the manufacturer’s recommendations. Furthermore, measuring the effects of exercise on ANS modulation hours after treatment would have provided valuable insights into the delayed effects of the intervention. Moreover, the lack of blinding could influence participant and researcher expectations. The absence of a control group made it challenging to differentiate the effects of the interventions from other external factors. Future studies should consider incorporating blinding and control groups to mitigate these biases.

### 4.3. Future Lines of Research

Further research on ANS modulation in fibromyalgia patients is essential, particularly regarding the effects of the EXOPULSE Mollii suit. As this is a novel therapeutic treatment, it still requires extensive exploration in this disease. Additionally, the effects of exercise on ANS modulation should be studied further, extending measurements to occurring both during the intervention, to better understand the response to exertion in fibromyalgia patients, as well as several hours post-treatment to explore potential delayed effects. Furthermore, future research should include a follow-up assessment after treatment to shed light on the lasting effects of both treatments.

## 5. Conclusions

In conclusion, both treatments can aid in ANS modulation in fibromyalgia patients. The neuromodulation treatment with the EXOPULSE Mollii suit showed greater beneficial effects on cortical arousal, microcirculation, and HRV whereas the Exercise intervention only demonstrated beneficial effects on cortical arousal. However, further research is needed for both treatments to better understand their full potential and long-term impact on fibromyalgia management.

## Figures and Tables

**Table 1:** Cortical arousal and thermography descriptive statistics.

Variables	Suit						Exercise					
	1st Session		8th Session		16th Session		1st Session		8th Session		16th Session	
	Pre	Post	Pre	Post	Pre	Post	Pre	Post	Pre	Post	Pre	Post
Cortical Arousal, Hz	32.00 ± 2.39	32.50 ± 2.48	31.10 ± 3.38	32.20 ± 2.46	31.70 ± 2.91	33.10 ± 2.39	32.00 ± 2.48	33.50 ± 3.17	31.60 ± 2.86	32.80 ± 2.92	28.80 ± 10.52	32.60 ± 2.97
Palm Tª, °C	31.50 ± 4.65	30.90 ± 3.25	33.40 ± 2.63	33.00 ± 3.19	34.90 ± 1.75	33.40 ± 3.15	32.30 ± 3.17	34.50 ± 2.16	34.30 ± 1.99	35.70 ± 0.84	34.90 ± 1.83	36.20 ± 1.10
Backhand Tª, °C	30.70 ± 4.56	30.60 ± 3.41	32.40 ± 3.12	32.20 ± 2.74	33.90 ± 2.18	32.90 ± 3.47	31.80 ± 3.52	33.70 ± 2.58	33.30 ± 2.45	34.60 ± 1.08	34.60 ± 1.64	35.40 ± 1.11
Distal Index finger Tª, °C	28.90 ± 5.73	28.80 ± 3.83	29.30 ± 3.77	28.30 ± 3.37	32.00 ± 2.15	27.80 ± 3.74	30.50 ± 5.10	33.20 ± 3.55	31.10 ± 3.73	32.70 ± 2.51	32.10 ± 2.50	34.10 ± 2.35
Proximal Index finger Tª, °C	30.00 ± 5.94	29.40 ± 4.07	32.50 ± 3.45	31.30 ± 4.01	34.00 ± 2.59	31.80 ± 4.56	31.00 ± 4.99	34.00 ± 3.20	33.80 ± 2.63	35.10 ± 0.89	34.50 ± 2.46	36.00 ± 1.10

Data are presented as means ± SDs. Tª: Temperature.

**Table 2:** HR and HRV descriptive statistics.

Variables	Suit						Exercise					
	1st Session		8th Session		16th Session		1st Session		8th Session		16th Session	
	Pre	Post	Pre	Post	Pre	Post	Pre	Post	Pre	Post	Pre	Post
Mean HR, bpm	79.75 ± 9.99	74.12 ± 8.33	77.48 ± 7.84	74.68 ± 7.44	81.46 ± 7.95	84.36 ± 21.60	77.53 ± 7.64	79.61 ± 6.33	76.65 ± 11.29	84.53 ± 12.10	78.50 ± 5.19	90.78 ± 10.69
Max. HR, bpm	99.71 ± 24.57	92.65 ± 22.00	90.10 ± 10.43	91.66 ± 16.62	100.93 ± 20.74	97.25 ± 18.68	91.45 ± 12.53	95.64 ± 7.90	89.12 ± 9.55	101.86 ± 13.73	97.69 ± 9.73	110.96 ± 14.09
Min. HR, bpm	68.17 ± 8.92	64.15 ± 7.94	68.70 ± 7.71	65.41 ± 5.69	70.77 ± 7.57	69.52 ± 11.77	69.29 ± 6.30	69.76 ± 7.08	69.52 ± 11.87	74.96 ± 11.90	68.86 ± 4.65	79.11 ± 7.75
RMSDD, ms	17.59 ± 5.89	18.74 ± 2.89	17.83 ± 4.64	19.15 ± 4.73	17.06 ± 3.92	17.09 ± 3.07	17.16 ± 4.03	15.23 ± 5.35	17.30 ± 6.11	13.51 ± 5.48	15.16 ± 4.42	12.11 ± 5.49
PNN50, %	1.71 ± 2.00	1.60 ± 1.10	1.51 ± 1.49	3.96 ± 6.48	0.94 ± 1.02	1.33 ± 1.14	1.44 ± 1.22	0.83 ± 1.27	1.35 ± 1.22	0.66 ± 0.77	10.29 ± 29.12	0.75 ± 1.17
HF, n.u.	26.48 ± 21.14	20.91 ± 11.97	26.75 ± 16.83	23.64 ± 11.89	18.99 ± 12.69	15.99 ± 5.26	25.14 ± 12.57	17.33 ± 8.39	23.87 ± 9.98	16.81 ± 10.70	14.04 ± 4.92	13.64 ± 9.01
LF, n.u.	73.47 ± 21.18	79.04 ± 12.01	71.70 ± 17.63	76.31 ± 11.90	80.96 ± 12.73	83.99 ± 5.27	74.81 ± 12.58	82.64 ± 8.40	76.05 ± 10.02	83.15 ± 10.71	85.92 ± 4.92	86.35 ± 9.02
SD1, ms	12.44 ± 4.17	13.26 ± 2.05	12.61 ± 3.28	13.55 ± 3.35	12.07 ± 2.78	12.09 ± 2.18	12.14 ± 2.85	10.77 ± 3.78	12.24 ± 4.32	9.56 ± 3.88	10.80 ± 3.07	8.57 ± 3.88
SD2, ms	36.21 ± 12.07	37.86 ± 10.64	31.93 ± 7.43	37.69 ± 7.09	38.53 ± 12.84	43.14 ± 16.96	30.88 ± 5.94	33.27 ± 8.16	32.07 ± 9.46	28.96 ± 9.76	35.07 ± 7.43	30.86 ± 8.78

Data are presented as means ± SDs. HR: Heart Rate. RMSSD: Root Mean Square of Successive Differences. PNN50: percentage of successive normal sinus (R-R) intervals that differed by more than 50 milliseconds. LF: Low frequency. HF: High frequency. n.u.: Normalized unit. SD1: Poincaré plot index of short-term variability in heart rate. SD2: Poincaré plot index of long-term variability in heart rate.

**Table 3:** One-way repeated-measures ANOVA results.

Variables	Suit		Exercise	
	Pre (*p*)	Post (*p*)	Pre (*p*)	Post (*p*)
Cortical Arousal, Hz	0.741	0.301	0.741	0.641
Palm Tª, °C	0.006 *	0.05 *	0.023 *	0.045 *
Backhand Tª, °C	0.003 *	0.135 *	0.032 *	0.045 *
Distal Index finger Tª, °C	0.122	0.69	0.819	0.236
Proximal Index finger Tª, °C	0.045 *	0.062 *	0.042 *	0.05
Mean HR, bpm	0.045 *	0.641	0.093	0.008 *
Max. HR, bpm	0.121	0.325	0.135	0.002 *
Min. HR, bpm	0.121	0.549	0.223	0.008 *
RMSDD, ms	0.717	0.717	0.135	0.223
PNN50, %	0.131	0.459	0.135	0.483
HF, n.u.	0.459	0.169	0.03 *	0.417
LF, n.u.	0.325	0.169	0.03 *	0.417
SD1, ms	0.717	0.717	0.135	0.223
SD2, ms	0.236	0.459	0.882	0.607

Table presents one-way repeated-measures ANOVA results comparing all baseline and post-session measurements within one. * *p* < 0.05. Tª: Temperature. HR: Heart Rate. RMSSD: Root Mean Square of Successive Differences. PNN50: percentage of successive normal sinus (R-R) intervals that differed by more than 50 milliseconds. LF: Low frequency. HF: High frequency. n.u.: Normalized unit. SD1: Poincaré plot index of short-term variability in heart rate. SD2: Poincaré plot index of long-term variability in heart rate.

**Table 4:** Between-group comparative statistics.

Variables	Pre 1st Session	Post 1st Session	Pre 8th Session	Post 8th Session	Pre 16th Session	Post 16th Session
	ε^2^	ε^2^	ε^2^	ε^2^	ε^2^	ε^2^
Cortical Arousal, Hz	0.002	0.019	0.003	0.019	0.001	0.037
Palm Tª, °C	0.000	0.348 *	0.037	0.197	0.000	0.203
Backhand Tª, °C	0.027	0.205	0.022	0.230 *	0.011	0.111
Distal Index finger Tª, °C	0.043	0.253 *	0.051	0.358 *	0.013	0.612 *
Proximal Index finger Tª, °C	0.008	0.299 *	0.040	0.220 *	0.000	0.243 *
Mean HR, bpm	0.006	0.072	0.026	0.193 *	0.026	0.125
Max. HR, bpm	0.026	0.125	0.026	0.140	0.006	0.209
Min. HR, bpm	0.006	0.084	0.001	0.275 *	0.019	0.373 *
RMSDD, ms	0.006	0.125	0.000	0.232 *	0.041	0.212
PNN50, %	0.004	0.176	0.003	0.276 *	0.004	0.143
HF, n.u.	0.001	0.014	0.000	0.072	0.033	0.019
LF, n.u.	0.001	0.014	0.002	0.072	0.033	0.019
SD1, ms	0.006	0.125	0.000	0.232 *	0.041	0.212
SD2, ms	0.026	0.041	0.009	0.212	0.006	0.212

Table presents comparisons between both treatments for each measure. * *p* < 0.05. Tª: Temperature. HR: Heart Rate. RMSSD: Root Mean Square of Successive Differences. PNN50: percentage of successive normal sinus (R-R) intervals that differed by more than 50 milliseconds. LF: Low frequency. HF: High frequency. n.u.: Normalized unit. SD1: Poincaré plot index of short-term variability in heart rate. SD2: Poincaré plot index of long-term variability in heart rate.

**Table 5:** Intrasession comparative statistics.

	Suit			Exercise		
Variables	1st Session	8th Session	16th Session	1st Session	8th Session	16th Session
	Effect Size	Effect Size	Effect Size	Effect Size	Effect Size	Effect Size
Cortical Arousal, Hz	0.540	0.727 *	0.972 *	1.043 *	0.747 *	0.600
Palm Tª, °C	−0.211	−0.230	−0.694	0.819 *	0.861 *	0.689
Backhand Tª, °C	−0.038	−0.099	−0.325	0.769 *	0.756	0.609
Distal Index finger Tª, °C	−0.016	−0.423	−1.499 *	0.636	0.379	0.916 *
Proximal Index finger Tª, °C	−0.164	−0.627	−0.592	−0.948 *	−0.876 *	−0.5 *
Mean HR, bpm	−0.835 *	−0.660	−0.111	0.644	0.867 *	1 *
Max. HR, bpm	−0.422	0.094	−0.389	0.625	0.911 *	1.123 *
Min. HR, bpm	−0.595	−0.923 *	−0.467	0.111	0.867 *	1.432 *
RMSDD, ms	0.2845	0.362	0.008	−0.740	−1.028 *	−0.698 *
PNN50, %	−0.067	0.333	0.467	−0.679	−0.859 *	−0.048
HF, n.u.	−0.345	−0.23	−0.267	−1.127 *	−0.681	−0.058
LF, n.u.	0.345	0.3	0.269	1.127 *	0.682	0.062
SD1, ms	0.285	0.363	0.008	−0.736	−1.027 *	−0.718
SD2, ms	0.196	0.957 *	0.067	0.557	−0.502	−0.721

Table presents *t*-test results comparing baseline and post-session measurement for each session and treatment. * *p* < 0.05. Tª: Temperature. HR: Heart Rate. RMSSD: Root Mean Square of Successive Differences. PNN50: percentage of successive normal sinus (R-R) intervals that differed by more than 50 milliseconds. LF: Low frequency. HF: High frequency. n.u.: Normalized unit. SD1: Poincaré plot index of short-term variability in heart rate. SD2: Poincaré plot index of long-term variability in heart rate.

**Table 6:** Suit treatment intersession comparative statistics.

Variables	1st vs. 8th Pre Session	8th vs. 16th Pre Session	1st vs. 16th Pre Session	1st vs. 8th Post Session	8th vs. 16th Post Session	1st vs. 16th Post Session	1st Pre vs. 8th Post Session	1st Pre vs. 16th Post Session	8th Pre vs. 16th Post Session
	Effect Size	Effect Size	Effect Size	Effect Size	Effect Size	Effect Size	Effect Size	Effect Size	Effect Size
Cortical Arousal, Hz	0.399	−0.192	0.123	0.361	−0.534	−0.434	−0.350	−0.616	−0.8 *
Palm Tª, °C	−0.757 *	−1.12 *	−1.03 *	−0.653	−0.342	−0.85 *	−0.471	−0.592	−0.0782
Backhand Tª, °C	−1.25 *	−0.77 *	−1.25 *	−0.6	−0.402	−0.73	−0.69	−0.929 *	−0.273
Distal Index finger Tª, °C	−0.106	−0.9*	−0.76*	0.142	−0.0308	0.389	0.178	0.206	0.555
Proximal Index finger Tª, °C	−0.782 *	−0.93 *	−0.98 *	−0.51	−0.299	−0.485	−0.341	−0.413	0.128
Mean HR, bpm	0.25	−0.97 *	−0.157	−0.0554	−0.467	−0.289	0.509	0.0222	−0.2
Max. HR, bpm	0.422	−0.644	−0.378	0.0222	−0.722	−0.278	0.333	−0.556	−0.722
Min. HR, bpm	−0.062	−0.546	−0.415	−0.168	−0.333	−0.333	0.414	0.0667	0.0667
RMSDD, ms	−0.083	0.372	0.117	−0.0991	0.433	0.437	−0.391	0.0828	0.178
PNN50, %	0.056	0.756	0.667	−0.289	0.378	0.215	−0.156	0.0667	0.178
HF, n.u.	−0.026	0.675	0.466	−0.239	0.78 *	0.409	0.141	0.554	0.7
LF, n.u.	0.105	−0.782	−0.465	0.238	−0.78 *	−0.41	−0.141	−0.554	−0.823
SD1, ms	−0.082	0.373	0.118	−0.0988	0.433	0.438	−0.392	0.0831	0.178
SD2, ms	0.35	−0.6	−0.289	0.0131	0.0222	−0.0222	−0.142	−0.2	−0.6

Table presents *t*-test results comparing baseline and post-session measurements between different sessions within the Suit treatment. * *p* < 0.05. Tª: Temperature. HR: Heart Rate. RMSSD: Root Mean Square of Successive Differences. PNN50: percentage of successive normal sinus (R-R) intervals that differed by more than 50 milliseconds. LF: Low frequency. HF: High frequency. n.u.: Normalized unit. SD1: Poincaré plot index of short-term variability in heart rate. SD2: Poincaré plot index of long-term variability in heart rate.

**Table 7:** Exercise treatment intersession comparative statistics.

Variables	1st vs. 8th Pre Session	8th vs. 16th Pre Session	1st vs. 16th Pre Session	1st vs. 8th Post Session	8th vs. 16th Post Session	1st vs. 16th Post Session	1st Pre vs. 8th Post Session	1st Pre vs. 16th Post Session	8th Pre vs. 16th Post Session
	Effect Size	Effect Size	Effect Size	Effect Size	Effect Size	Effect Size	Effect Size	Effect Size	Effect Size
Cortical Arousal, Hz	0.373	0.127	0.2	0.444	0.0756	0.338	−0.645	−0.207	−0.365
Palm Tª, °C	−0.556	−0.464	−1 *	−0.73 *	−0.444	−0.686	−1 *	−1.26 *	−1.62 *
Backhand Tª, °C	−0.354	−0.626	−0.91 *	−0.145	−0.733	−0.802 *	−0.89 *	−1.22 *	−1.01 *
Distal Index finger Tª, °C	−0.0222	−0.513	−0.361	0.0941	−0.528	−0.283	−0.385	−0.683	−1.15 *
Proximal Index finger Tª, °C	−0.527	−0.444	−0.82 *	−0.2	−0.96 *	−0.822 *	−1 *	−1 *	−1.13 *
Mean HR, bpm	0.389	−1 *	−0.133	−0.444	−1.32 *	−0.956 *	−0.631	−1.21 *	−1 *
Max. HR, bpm	0.389	−0.94 *	−0.577	−0.362	−1.28 *	−1.43 *	−0.555	−1 *	−1 *
Min. HR, bpm	0.333	−0.667	0.072	−0.444	−1.24 *	−0.956 *	−0.52	−1.1 *	−1 *
RMSDD, ms	−0.487	1.06 *	0.559	0.0933	0.905 *	0.775 *	0.886 *	1.22 *	1.17 *
PNN50, %	−0.0433	0.444	0.556	−0.333	0.00885	0.333	0.553	0.65	0.458
HF, n.u.	−0.215	1.36 *	1.05	−0.0587	0.372	0.519	0.905 *	1.22 *	1.18 *
LF, n.u.	0.218	−1.35 *	−1.05	0.0602	−0.376	−0.52	−0.91 *	−1.22 *	−1.18 *
SD1, ms	−0.484	1.01 *	0.506	0.0929	0.905 *	0.775 *	0.886 *	1.22 *	1.17 *
SD2, ms	−0.392	−0.066	−0.562	0.313	0.067	0.392	−0.015	0.00346	0.401

Table presents *t*-test results comparing baseline and post-session measurements between different sessions within the Exercise treatment. * *p* < 0.05. Tª: Temperature. HR: Heart Rate. RMSSD: Root Mean Square of Successive Differences. PNN50: percentage of successive normal sinus (R-R) intervals that differed by more than 50 milliseconds. LF: Low frequency. HF: High frequency. n.u.: Normalized unit. SD1: Poincaré plot index of short-term variability in heart rate. SD2: Poincaré plot index of long-term variability in heart rate.

## Data Availability

All the data are present in the article.
